# The global landscape of pediatric monoclonal antibody (mAb) clinical trials: an analysis of ClinicalTrials.gov

**DOI:** 10.3389/fped.2026.1846142

**Published:** 2026-09-01

**Authors:** Agrata Sharma, Sanjana Mukherjee, Sharonann Lynch

**Affiliations:** Center for Global Health Policy and Politics, Georgetown University, Washington, DC, United States

**Keywords:** clinical trials, equity, global landscape, monoclonal antibodies, pediatric populations

## Abstract

**Introduction:**

Monoclonal antibodies (mAbs) are a growing class of biologics used to address many diseases, including cancers, autoimmune diseases, and infectious diseases, with several mAbs specifically approved for use in children for various indications. However, developing and using mAbs in pediatric populations is particularly challenging due to limited clinical trial data in children, which stems from recruitment difficulties, ethical considerations, and other issues. Furthermore, inconsistent access to clinical trials, especially in low- and middle-income countries (LMICs), hinders the widespread accessibility and applicability of mAbs. To highlight challenges and opportunities, this study reviews the global pediatric mAb clinical trials data.

**Methods:**

Using a cross-sectional design, data were extracted from ClinicalTrials.gov, the largest clinical trials database, managed by the U.S. National Library of Medicine (NLM) at the National Institutes of Health (NIH). Clinical trials were analyzed by geographic location, country income classification, and trial characteristics such as sponsor type and reasons for termination.

**Results:**

Our analysis identified 1,863 mAb clinical trials registered on ClinicalTrials.gov that permitted pediatric enrollment, of which only 239 (12.8%) exclusively enrolled pediatric populations. Most trials were conducted in high-income countries in the Global North, with trials in high-income countries outnumbering those in low-income countries by more than 200-fold, revealing significant inequities in clinical trial participation by geographic region. Of all mAb trials permitting pediatric enrollment, only 168 (9.0%) targeted an infectious disease indication; bacterial, fungal, and neglected tropical diseases were particularly underrepresented. Low patient accrual and trial attrition were the most commonly cited reasons for trial termination. Our results also revealed a significant transparency gap: fewer than one third of trials posted results, and only a small fraction made study protocols, statistical analysis plans, and informed consent forms publicly available, despite mandatory disclosure requirements.

**Discussion:**

The findings suggest that mAb clinical trials permitting pediatric enrollment are geographically concentrated, disease-selective, and insufficiently transparent. These findings can inform policy and investment decisions to ensure equitable and transparent research on mAbs for pediatric populations.

## Introduction

Widely considered a game-changer for disease prevention and treatment, monoclonal antibodies (mAbs) have revolutionized treatment and care for diseases including cancer, autoimmune diseases and infectious diseases. In pediatric populations, specifically, mAbs have been approved for use including nirsevimab and palivizumab for respiratory syncytial virus (RSV) lower respiratory tract disease, eculizumab for atypical haemolytic uremic syndrome, and brentuximab vedotin for previously untreated high risk classical Hodgkin lymphoma (1–4). Despite their immense clinical potential in pediatric populations, children may face delays in access to mAbs because clinical trials to determine safety and efficacy are often conducted only in adult populations. Furthermore, the lack of pediatric-specific clinical trial data has resulted in 38%–50% off-label use of many drugs and biologics, including mAbs, for treatment in children, which has been identified as a public health issue due to concerns regarding treatment failures and risk of adverse events (5–9).

Pediatric populations deserve medicines that are safe, effective, and underpinned by robust clinical evidence. Yet children have historically been termed “therapeutic orphans” and systematically excluded from drug development despite bearing a disproportionate share of the global disease burden. Bourgeois et al. found that while children accounted for 59.9% of the global disease burden, only 12% of clinical trials were pediatric (10). Further, estimates suggest that 50%–75% of medicines prescribed to children have not been tested in pediatric populations, and safety and efficacy data from studies conducted in adults are regularly extrapolated for use in children (11, 12). Several factors have hindered clinical trial studies in pediatric populations including challenges related to recruiting sufficient children and inability to secure an appropriate sample size for trial studies, lower economic returns on research investments for manufacturers due to a smaller pediatric medication market, and ethical considerations related to pediatric clinical trials (13, 14).

Furthermore, many regions remain underrepresented in pediatric clinical trials and there are significant global disparities in the global burden of disease and where clinical research efforts are concentrated, which significantly limits the generalizability of the knowledge generated by clinical studies. Estimates show that 43% of clinical trials are conducted in low- and middle-income countries (LMICs) despite almost 80% of the world's population living in LMICs (15). These disparities are also seen across dimensions of inequality such as low socioeconomic status and racial/ethnic minorities, who experience greater negative health outcomes but are severely underrepresented in clinical trials (16, 17).

Understanding the landscape of clinical trial data for pediatric populations is crucial to highlight opportunities to strengthen trial ecosystems and optimize the research process specifically for children. Clinical trial databases, such as ClinicalTrials.gov and the World Health Organization (WHO) International Clinical Trials Registry Platform (ICTRP), serve as an important repository on the current landscape of clinical research. Clinical trial transparency, through the availability of clinical trial information in such databases, is critical for research integrity and for advancing scientific research (18). Information on trial registration, publication of clinical study reports (CSRs) which provide detailed description on the design, study protocols, results, and statistical analyses of clinical trials, reasons for clinical trial termination and other clinical trial data can prevent duplication of efforts and help the scientific community, funders and other stakeholders identify research gaps. Furthermore, increased transparency of clinical trial data can strengthen public trust, improve accountability, and foster innovation.

By conducting a comprehensive review of the global pediatric mAbs pipeline, this study aims to identify key trends, gaps and disparities in mAbs clinical research for pediatric populations and increase transparency and accountability surrounding clinical trial data related to mAbs. Results from this study will provide robust evidence base for strategic policy formulation, health guideline development, and resource allocation decisions which are aligned to better address public health challenges specifically in pediatric populations.

## Methodology

### Study design

We designed a cross-sectional study to analyze the landscape of pediatric mAbs clinical trials registered on ClinicalTrials.gov until January 5, 2026. As it did not involve clinical data or human participants, this study was not submitted for ethics review.

### Data collection and extraction

Clinical trial data were obtained from ClinicalTrials.gov, a publicly accessible registry administered by the National Library of Medicine (NLM) at the National Institutes of Health (NIH). It is the largest clinical trials registry in the world, hosting privately and publicly funded studies conducted across multiple countries (19). The database was searched on January 5, 2026, using monoclonal antibodies as a Medical Subject Heading (MeSH) term– a controlled NLM vocabulary used to index biomedical and clinical study records–combined with the variations of search terms including “monoclonal antibodies,” “monoclonal antibody,” or “mAbs” (20). The resulting records were subsequently filtered to retain only those trials that included pediatric populations, identified by the presence of the term “child” in the age eligibility field.

### Data analysis and coding

Data were managed using MS Excel and all data processing was performed using R version 4.2.2. The following trial characteristics were extracted and analyzed: geographic classification, disease classification, terminated trials, sponsor, funder type, trial phase, type of clinical trial, trial status, and age eligibility.

ClinicalTrials.gov categorizes participant age eligibility into three standardized groups: “Child” (birth-17 years), “Adult” (18–64 years), and “Older Adult” (65 years and above). All trials designating the “Child” age group as an eligible population were included in the final dataset, irrespective of whether the trial also enrolled adult or older adult participants. No restrictions were placed on trial status, geographic location, disease condition, or date of registration, study start or end date, and both interventional and observational studies were eligible for inclusion. A total of 1,863 results were obtained. This inclusion criterion captures trials in which children were an eligible population rather than the exclusive or primary study population.

For geographical classification of clinical trial data, location data extracted from ClinicalTrials.gov were cleaned and standardized to the country level. Each trial was then categorized as either single-country or multi-country based on the number of distinct participating countries. Countries were further classified according to geographic regions and to income group designations (2025) based on the World Bank categorization. Venezuela was excluded from income group analysis as it remains unclassified in the World Bank database (Supplementary Methods).

Several variables not available in the standard downloaded dataset were added manually. For age, as ClinicalTrials.gov captures only the broad age group designations described above, the minimum and maximum participant age limits were manually extracted from individual trial records and appended to the dataset.

For disease classification, the “conditions” data were classified into two broad categories: infectious diseases and non-communicable diseases. Trials were manually classified as infectious if at least one condition under investigation had an infectious etiology, and were then assigned to a single pathogen-based subgroup (viral, chronic viral, bacterial, parasitic, or fungal) accordingly.

Study status classifications were derived from ClinicalTrials.gov-reported fields. These include “Completed” referred to studies that ended normally following completion of participant follow-up. Ongoing studies included “Recruiting,” “Not yet recruiting,” “Enrolling by invitation,” and “Active, not recruiting,” reflecting trials at different stages of enrollment or follow-up. Studies that were halted prematurely were further differentiated to preserve analytical granularity: “Suspended” indicated a temporary halt with potential for resumption; “Terminated” referred to studies stopped early with no plans to resume and with participants no longer receiving intervention or follow-up; and “Withdrawn” denoted studies stopped prior to enrollment of the first participant. Additional categories included “Available”, “No longer available”, “Approved for marketing,” and “Unknown” for records with missing or indeterminate status.

For trials recorded as terminated, the reason for termination as reported by the trial investigators was manually extracted and coded. These reasons were subsequently grouped into five categories: (1) Financial and Resource Constraints; (2) Logistic, Operational, and Administrative Reasons; (3) Scientific Reasons; (4) Strategic or Business Decisions; and (5) Unknown (Supplementary Methods). Studies were classified as “Unknown” if no reason was provided or if the reason for termination was unclear, vague or not properly defined. The focus of our analysis was on terminated trials as these trials were halted prematurely and would not resume.

To classify trials based on sponsor type, we extracted “Sponsor” data from ClinicalTrials.gov. The database defines “Sponsor” as the “the organization or person who initiates the study and who has authority and control over the study” (21). Further, the database records organizations listed as sponsors and/or collaborators for a study as the funders of the study and categorises it under funder type. The support they provide may vary to include support in terms of funding, design, implementation, data analysis, or reporting. The database categorizes the funders as: 1) U.S. National Institutes of Health (NIH); 2) other U.S. Federal agencies (for example, Food and Drug Administration, Centers for Disease Control and Prevention, or U.S. Department of Veterans Affairs); 3) industry (for example: pharmaceutical and device companies); 4) all others (including individuals, universities, and community-based organizations) (21). Two investigators independently coded the sponsor data to verify if the data were coded correctly in the database (Supplementary Data). While the ClinicalTrials.gov glossary defines “Other U.S. Federal agencies” as a separate group, in our analysis none of the trials that were classified as “Other Gov” were U.S. government funded but were funded by governments of other countries. The analysis also revealed a separate “Network” category which was not included in the glossary. To account for these inconsistencies, we reclassified funder types into the following five categories: 1) U.S. Government (NIH and Other U.S. Federal agencies); 2) other Governments (Government agencies and institutions of other countries) 3) industry; 4) networks (collection of organizations and research institutions that coordinate and support clinical trials); and 5) others.

All statistical analyses and data visualization were carried out using MS Excel and R version 4.2.2.

### Reproducibility and data availability

All data used in this analysis were derived from the publicly available ClinicalTrials.gov registry. The dataset, R analysis scripts, and coding dictionary for terminated trials are provided as Supplementary Material to support reproducibility. The manual coding of age data, conditions and termination reasons was performed by two investigators; discrepancies were resolved by consensus.

## Results

A total of 17,169 monoclonal antibody (mAb) trials have been registered to date in ClinicalTrials.gov, of which 1,863 (10.9%) included pediatric participants, though not necessarily as the exclusive or primary study population. Among these studies, 84% were interventional (*n* = 1,568), 15% were observational (*n* = 276), and 1% involved expanded access (*n* = 19).

### Characteristics of pediatric mAbs clinical trials by geographic location and income classification

**Figure 1:**
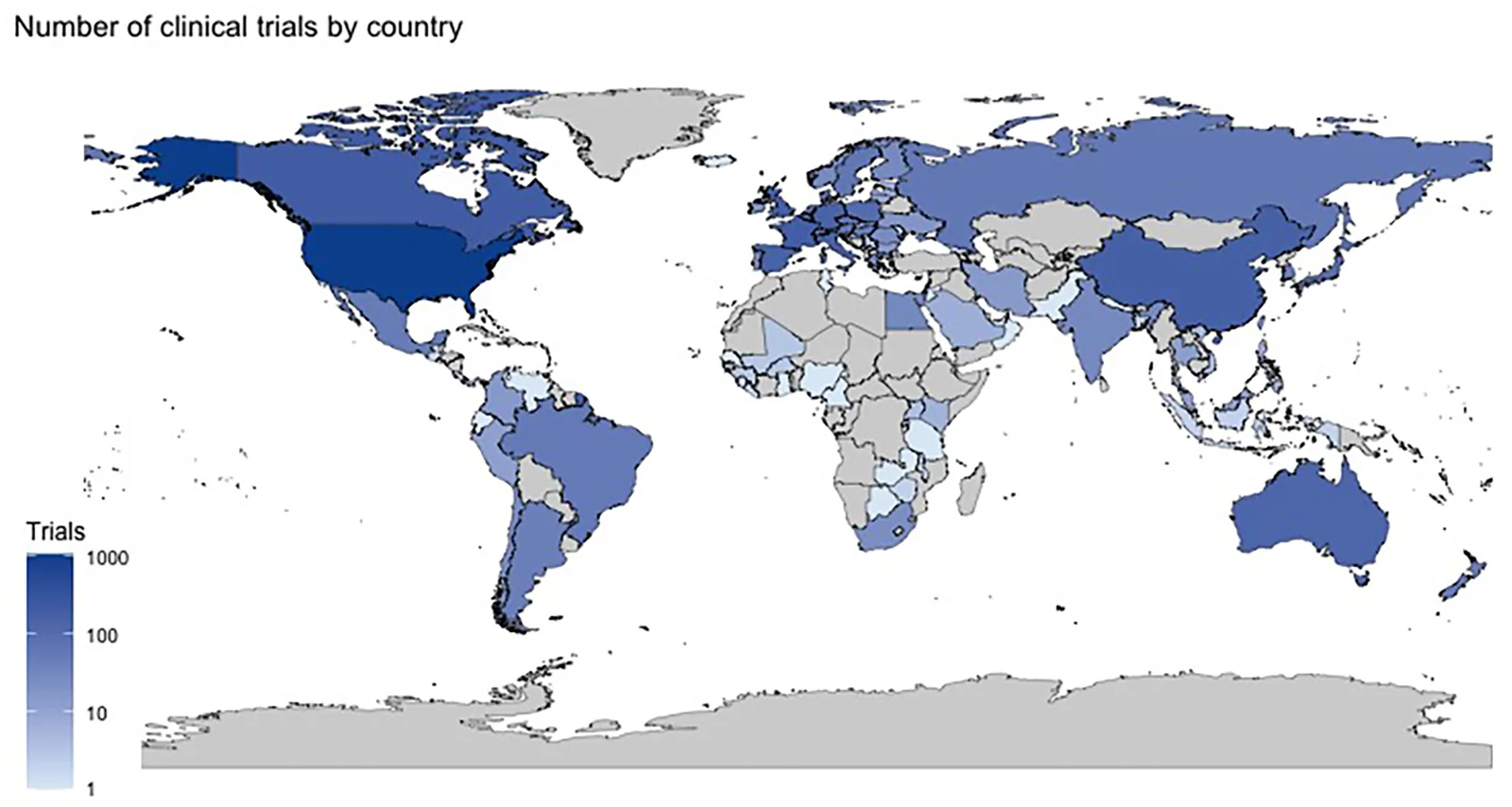
Number of pediatric monoclonal antibody clinical trials by country. The number of trials listed in ClinicalTrials.gov is reported by country until January 5, 2026. Highest concentration of clinical trials is represented by dark blue while lighter colors represent lower concentrations of clinical trials.

**Figure 2:**
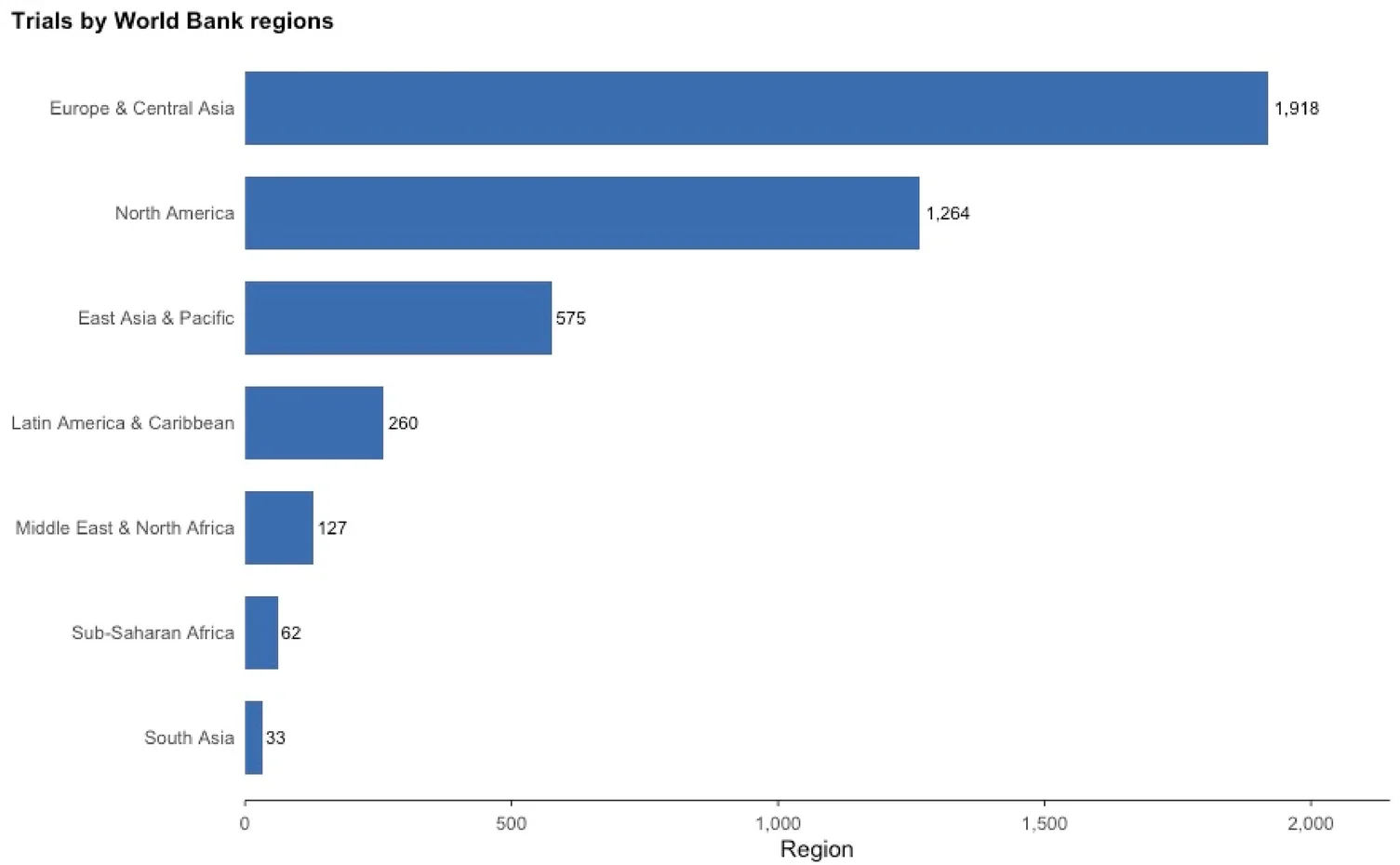
Geographic distribution of pediatric monoclonal antibody clinical trials by region. Countries were classified into regions based on the World Bank (WB) region classification. Each participating country is counted as a unique entry per trial; multiple sites within the same country are aggregated into a single country-level observation.

In terms of geographic distribution, the United States accounted for the largest share of trials (*n* = 1,061), representing a volume more than five times greater than that of the second-ranked country. Canada ranked second (*n* = 203), followed by France (*n* = 187), the United Kingdom (*n* = 179), China (*n* = 170), Italy (*n* = 164), Germany (*n* = 157), Spain (*n* = 154), Australia (*n* = 128), Belgium (*n* = 117), and Poland (*n* = 108). All remaining countries each contributed fewer than 100 trials. Further, “Locations” data were missing in the database for 161 trials (Figure 1). At the regional level, Europe and Central Asia (*n* = 1,981) and North America (*n* = 1,264) dominated the distribution of pediatric mAb trials, despite these regions collectively accounting for a relatively small proportion of the global pediatric population (22). Conversely, sub-Saharan Africa and South Asia, with the highest pediatric population, were the most underrepresented regions (Figure 2) (22).

**Figure 3:**
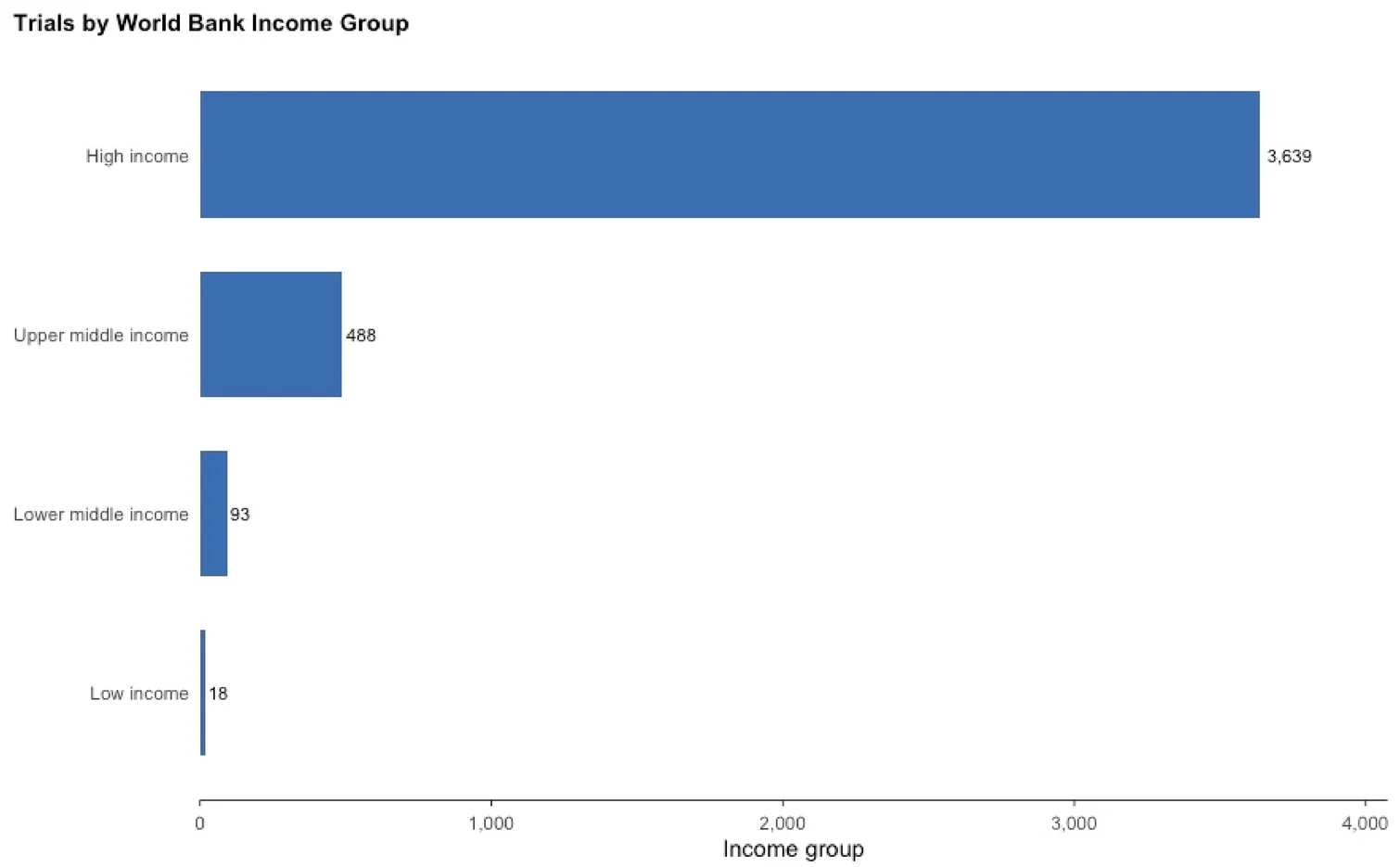
Number of pediatric monoclonal antibody clinical trials by income group. Countries were classified into four income groups based on the World Bank (WB) income classification. Each participating country is counted as a unique entry per trial. The total number of trials depicted in the figure exceeds the total 1,863 pediatric mAbs trials in our study as a trial can have multiple countries participating in the trial.

When analyzed by income group, the discrepancy is even greater. High-income countries accounted for the vast majority of pediatric mAb trials (*n* = 3,639), followed by upper-middle-income countries (*n* = 488), lower-middle-income countries (*n* = 93), and low-income countries (*n* = 18) (Figure 3).

Of the 1,863 pediatric mAb trials, “locations” data was available for 1,702 studies. A majority were conducted in a single country (*n* = 1,346, 79.1%), while 356 trials (20.9%) were multi-country in design. Among the multi-country trials, the United States again led the participation with 273 trials followed by Canada (*n* = 185), France (*n* = 148), the United Kingdom (*n* = 146), Germany (*n* = 144), Spain (*n* = 142), Italy (*n* = 132), Australia (*n* = 122), Belgium (*n* = 110), and Poland (*n* = 104). Most of the North American and European countries maintained a relatively high proportion of multi-country participation relative to their total trial counts. Nevertheless, China, which ranked fifth in overall trial count, contributed only 16 multi-country trials, indicating that the majority of Chinese mAb trials were conducted domestically. As with the overall distribution, representation from LMICs in multi-country trials remains low. Of the 356 multi-country trials, only 118 (33.1%) included countries from different income groups, indicating that the majority of multi-country trials were conducted exclusively within countries of the same income tier.

Further, only 5 trials did not include a high-income country among their participating sites, underscoring that international collaboration in pediatric mAbs research remains largely concentrated within and between high-income countries (Table 1). Notably, the study sponsors of these five trials were all based in high income countries.

**Table 1:** Pediatric monoclonal antibody clinical trials for infectious diseases conducted exclusively in low- and middle-income countries (LMICs).

NCT number	Study Title	Status	Condition(s)	Sponsor	Phase	Enrollment (n)	Funder Type	Country
NCT01196520	Epidemiology of Burkitt Lymphoma in East Africa Children or Minors (EMBLEM)	Completed	Non-Hodgkin lymphoma; Malaria; Epstein–Barr virus	National Cancer Institute (NCI)	—	4,893	NIH	Tanzania; Uganda
NCT02564523	Safety, Tolerability and Immunogenicity of Three Prime-Boost Regimens for Ebola Vaccines Ad26.ZEBOV/MVA-BN-Filo	Completed	Hemorrhagic fever, Ebola	Janssen Vaccines & Prevention B.V.	2	1,075	Industry	Burkina Faso; Côte d'Ivoire; Kenya; Uganda
NCT03208231	Safety and Antiviral Activity of Monoclonal Antibody VRC01 in Infants With HIV	Completed	HIV infections	NIAID	1/2	61	NIH	Botswana; Brazil; Malawi; Zimbabwe
NCT05845112	Start Taking Action for TB Diagnosis	Completed	Tuberculosis	Liverpool School of Tropical Medicine	—	14,747	Other	Bangladesh; Brazil; Cameroon; Kenya; Malawi; Nigeria; Vietnam
NCT06841614	EBOla Post-Exposure Prophylaxis	Not yet recruiting	Ebola virus disease	ANRS, Emerging Infectious Diseases	3	160	Govt.	Democratic Republic of Congo; Guinea; Liberia; Sierra Leone

### Sponsors and funders of pediatric mAbs clinical trials

ClinicalTrials.gov defines “Sponsor” as the “the organization or person who initiates the study and who has authority and control over the study.” The database provides details about the sponsor and the collaborators and then categorizes the sponsor into the “funder type” category in its dataset. Notably, there were misclassifications and inconsistencies in classification of funder type; we noted 26 such inconsistencies and misclassifications in our analysis. Many records in the database were classified as “Network” but are not defined in the ClinicalTrials.gov glossary (21). The “Network” category was retained separately in our analysis. Additionally, “Individuals” were inconsistently categorized either separately or under “Others”; in our analysis, we consistently reclassified all individuals under “Others”. The frequency of cleaned funder types included in the dataset are listed in Table 2. The majority (60.12%) of pediatric mAbs clinical trials were funded by others (individuals, universities, and community-based organizations), followed by industry (26.36%). Of the 176 clinical trials funded by the United States government, 175 were funded by the NIH while 1 was funded by other U.S. Federal agencies. Less than 1% of mAbs clinical trials were funded by government agencies of other countries. Of the 17 clinical trials funded by other governments, only 7 were funded by upper-middle income and lower-middle income countries; the rest were funded by high income countries.

**Table 2:** Distribution of pediatric monoclonal antibodies clinical trials by funder type.

Funder type	Number of trials (n)	Percent (%) trials
Other	1,120	60.12%
Industry	491	26.36%
U.S. government	176	9.45%
NIH	175	
Other U.S. federal agencies	1	
Networks	59	3.17%
Other governments	17	0.91%
Total	1,687	100%

Among the top 10 sponsors, studies were led by non-industry institutions funded by “Other,” with M.D. Anderson Cancer Center (*n* = 109) and Memorial Sloan Kettering Cancer Center (*n* = 51) as the largest contributors. NIH-funded trials also accounted for a substantial share, led by the National Cancer Institute (*n* = 101) and the National Institute of Allergy and Infectious Diseases (NIAID) (*n* = 41). Industry-funded contributed fewer studies overall, with Novartis Pharmaceuticals (*n* = 37), Bristol-Myers Squibb (*n* = 30), and Genentech, Inc. (*n* = 23) representing the top pharmaceutical sponsors. The list of the top 10 sponsors is presented in Table 3.

**Table 3:** List of the top 10 sponsors for pediatric clinical trials.

Sponsors	Funder type	Count
M.D. Anderson Cancer Center	Other	109
National Cancer Institute (NCI)	U.S. Government (NIH)	101
Memorial Sloan Kettering Cancer Center	Other	51
National Institute of Allergy and Infectious Diseases (NIAID)	U.S. Government (NIH)	41
Novartis Pharmaceuticals	Industry	37
St. Jude Children's Research Hospital	Other	31
Baylor College of Medicine	Other	31
Bristol-Myers Squibb	Industry	30
Genentech, Inc.	Industry	23
Masonic Cancer Center, University of Minnesota	Other	22

### Distribution of pediatric mAbs clinical trials by disease

Of the 1,863 trials, 168 (9.0%) included at least one condition with an infectious etiology and 1,693 (90.8%) did not; 2 trials could not be classified because no condition was specified. The most commonly studied compounds include rituximab (*n* = 284), bevacizumab (*n* = 155), alemtuzumab (*n* = 130), nivolumab (*n* = 102), infliximab (*n* = 88), omalizumab (*n* = 87), adalimumab (*n* = 55), ipilimumab (*n* = 49), brentuximab vedotin (*n* = 49), and cetuximab (*n* = 36). When any of these drugs were utilized in combination with other biologics or pharmaceuticals or in different dosages within the same trial, they were included in the overall total.

To identify trials that included infectious diseases, studies were manually identified through keyword screening of four fields: Conditions, Brief Summary, Study Title, and Interventions. A total of 168 trials were identified. Included studies were classified into five categories: Viral (*n* = 92, 54.7%), Chronic Viral (*n* = 46, 27.3%), Bacterial (*n* = 16, 9.5%), Parasitic (*n* = 11, 6.5%), and Fungal (*n* = 3, 1.7%). For viral conditions, the proportion of trials focused on RSV (*n* = 45, 48.91%) was the highest, followed by COVID-19 (*n* = 34, 36.95%), Ebola (*n* = 5, 5.43%), Dengue (*n* = 2, 2.17%), Rabies (*n* = 1, 1.08%), West Nile Fever (*n* = 1, 1.08%), and other (*n* = 1, 1.08%). When stratified by funder type, Industry was the most common funder (*n* = 46, 50%), with 28/45 trials (62%) funded by industry, compared to 14 of the 34 trials for COVID-19 (41%).

For RSV, several biologics were noted, including nirsevimab, palivizumab, motavizumab (MEDI-524), TNM001 (Retavibart), suptavumab, and clesrovimab. Similarly, for COVID-19, several biologics were employed including etesevimab (JS016), sotrovimab, bebtelovimab, casirivimab/imdevimab, bamlanivimab, tixagevimab/cilgavimab (Evusheld/AZD7442), Pemivibart (VYD222), and STI-9199 (COVISHEILD).

For chronic viral conditions, Epstein–Barr virus (EBV)-associated disease and post-transplant lymphoproliferative disorders (PTLD)—a lymphoproliferative disorder affecting people with EBV infection, constituted the largest chronic viral subgroup (*n* = 16, 34.78%), followed by HIV/AIDS (*n* = 11, 23.91%), Human papillomavirus (HPV) (*n* = 4, 8.69%), Hepatitis C (HCV) (*n* = 3, 6.52%), Cytomegalovirus (CMV) (*n* = 2, 4.34%), HSV (*n* = 2, 4.34%), Human T-lymphotropic virus (*n* = 2, 4.34%), Human polyomavirus 2 (*n* = 2, 4.34%), Hepatitis B Virus (HBV) (*n* = 1, 2.17%), and Kaposi sarcoma-associated herpesvirus (*n* = 1, 2.17%) (23).

Particularly, three interventional trials were captured that focused on HIV broadly neutralising antibodies (bNAbs): NCT02256631 (completed; Phase 1), NCT03208231(completed; Phase 1|Phase 2), and NCT06517693 (not yet recruiting; Phase 1). All three studies were sponsored by NIAID (funder type: U.S. government NIH). Key interventions include VRC01, VRC01LS, VRC07-523LS, PGT121.414.LS. The trials are being conducted in Botswana, Brazil, Malawi, Kenya, South Africa, United States, and Zimbabwe.

The funding landscape for chronic viral trials differed substantially from the acute viral category: industry funding was almost absent (*n* = 3, 7%), with the majority funded through other sources (*n* = 27, 59%) or U.S. government NIH (*n* = 12, 26%). HIV/AIDS trials were overwhelmingly U.S. government NIH-funded (*n* = 9, 82%).

Of the 11 trials that focused on parasitic conditions, clinical trials for malaria mAbs were the most common (*n* = 9, 82%), with single trials each for visceral leishmaniasis and cysticercosis/helminthiasis. All 11 parasitic trials were funded by non-industry sources: U.S. government NIH (*n* = 4, 36%) and other (*n* = 7, 64%). Key mAbs interventions included broadly neutralising antibodies targeting *Plasmodium* species (TB31F, L9LS, MAM01 alongside vaccines like RH5.1 and R21). All 11 parasitic trials are conducted in LMICs. For malaria, trials were conducted in Mali (*n* = 3), Kenya (*n* = 2), Burkina Faso (*n* = 2), Uganda (*n* = 2), and Tanzania (*n* = 1). The clinical trial for visceral leishmaniasis was conducted in Kenya while the trial for cysticercosis/helminthiasis was conducted in Zambia.

Of the 3 trials addressing fungal conditions, one focused on allergic bronchopulmonary aspergillosis (ABPA) in the context of cystic fibrosis and two focused on the treatment of fungal infections among children with granulocytopenia and fever in the setting of cancer.

### Key characteristics of pediatric mAbs clinical trials by age eligibility

The ClinicalTrials.gov data does not provide details regarding average age of enrollment in the clinical trials. It only shares the inclusion and exclusion criteria based on age. Out of the 1,863 trials, 1,102 (59.15%) included “Child, Adult, Older Adult”, 522 (28.01%) included “Child, Adult” and only 239 (12.8%) included only “Child.”

The inclusion and exclusion age was added manually. For 405 trials the inclusion age was not specified and gave only the broad classification. The average age of inclusion of the mAbs clinical trials for the pediatric population was approximately 6.3 years (*n* = 1,458) while the cut off age was 40.1 years (*n* = 1,082). The average gap between the start age and the end age is approximately 35.3 years.

### Study status of pediatric mAbs clinical trials and key characteristics of terminated trials

Study status was classified as completed (*n* = 931, 49.97%), recruiting (*n* = 251, 13.47%), terminated (*n* = 199, 10.68%), unknown (*n* = 186, 9.98%), active but not recruiting (*n* = 156. 8.37%), withdrawn (*n* = 61, 3.27%), not yet recruiting (*n* = 44), no longer available (*n* = 11, 0.59%), enrolling by invitation (*n* = 9, 0.48%), suspended (*n* = 7, 0.37%), approved for marketing (*n* = 4, 0.21%), and available (*n* = 4, 0.21%).

**Figure 4:**
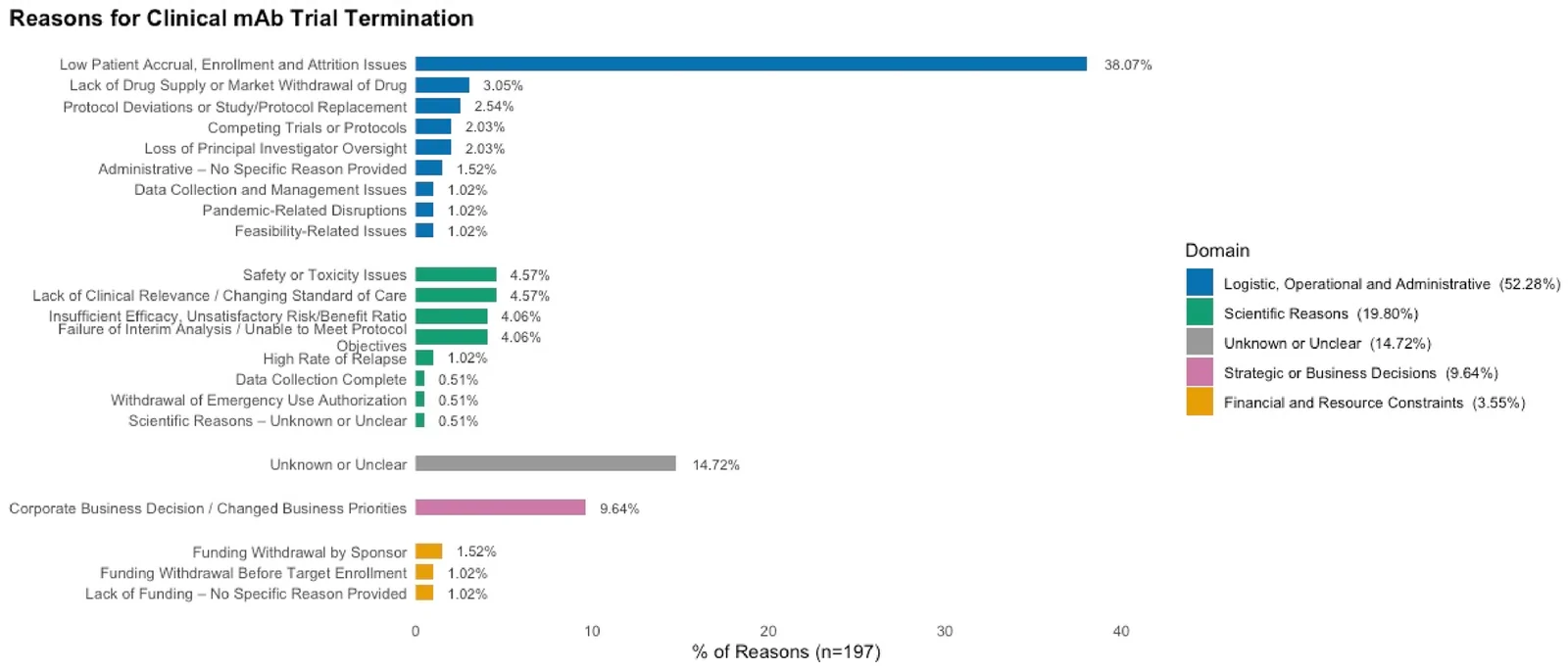
Reasons for termination of pediatric monoclonal antibody clinical trials. Our analysis identified 197 reasons why trials were terminated; two clinical trials had more than one reason for why the trial was terminated. **(A)** Reasons for trial termination by 5 major domains: (1) Financial and Resource Constraints; (2) Logistic, Operational, and Administrative Reasons; (3) Scientific Reasons; (4) Strategic or Business Decisions; and (5) Unknown. **(B)** Detailed reasons for trial termination within each domain.

Among terminated studies (*n* = 199), the vast majority were interventional (*n* = 191, 95.97%), with only a small proportion classified as observational (*n* = 8, 4.02%). The causes of the termination were classified into scientific reasons, strategic or business decisions, logistic, operational and administrative reasons, financial and resource constraints and unknown/unclear and then further into detailed reasons for terminations (Figure 4). Of the 199 terminated trials that were marked “Terminated”, the reason for termination for four trials were listed as “Study Complete”; these four studies were excluded from downstream analysis on reasons for trial termination. Our analysis of the 195 terminated trials identified 197 reasons why trials were terminated; two clinical trials had more than one reason for why the trial was terminated.

The majority of reasons why a trial was terminated was because of logistic, operational and administrative reasons (*n* = 103, 52.28%), followed by scientific reasons (*n* = 39, 19.8%). The largest portion of termination reasons were related to low patient accrual, enrollment and attrition issues (*n* = 75, 38.07%) followed by corporate business decisions and changes in business priorities (*n* = 19, 9.64%); 14.72% (*n* = 29) of all clinical trial termination reasons were classified as unknown or unclear as no reason was provided or the reason for termination was unclear, vague or not properly defined.

### Funder-specific patterns in pediatric mAbs clinical trial termination

The reasons for trial termination were classified based on “funder type”. Among industry-funded trials, terminations were relatively evenly distributed across scientific (*n* = 20), strategic or business (*n* = 19), and logistic, operational, and administrative domains (*n* = 18). Strategic considerations were particularly prominent, with corporate business decisions and changes in business priorities (*n* = 19) representing the most frequent subdomain. Operational challenges such as low patient accrual (*n* = 12), scientific drivers, including lack of clinical relevance or changes in standard of care (*n* = 8), as well as insufficient efficacy and interim analysis failures (*n* = 8 combined), also contributed to trial terminations.

In contrast, non-industry funded trials (U.S. government—NIH and other U.S. federal agencies, networks, other governments, and others) were predominantly terminated due to logistic, operational, and administrative reasons (*n* = 39), with low patient accrual, enrollment, and attrition issues accounting for the majority (*n* = 27). Scientific reasons (*n* = 9) and unknown or unclear reasons (*n* = 9) were less frequent, while financial and resource constraints (*n* = 4) and trials classified as study complete (*n* = 3) accounted for a small proportion. Additional factors included drug supply issues, safety or toxicity concerns, and protocol-related challenges (Table 4).

**Table 4:** Funder-specific patterns in pediatric mAbs clinical trial termination.

Domain	Subdomain	Industry (N)	Non-industry (N)	Domain total
Financial and resource constraints	Funding withdrawal before target enrollment	0	2	7
	Funding withdrawal by sponsor	0	3	
	Lack of funding (unspecified)	0	2	
Logistic, operational, and administrative reasons	Competing trials or protocols	1	3	103
	Data collection and management issues	1	1	
	Lack of drug supply or market withdrawal	0	6	
	Loss of principal investigator oversight	0	4	
	Low patient accrual, enrollment, attrition	13	62	
	Protocol deviations or study replacement	0	5	
	Pandemic-related disruptions	0	2	
	Feasibility-related issues	1	1	
	Administrative (unspecified)	3	0	
Scientific reasons	Insufficient Efficacy, Unsatisfactory Risk/Benefit Ratio	4	4	39
	Failure of Interim Analysis or Inability to Meet Protocol Objectives	4	4	
	Safety or toxicity issues	3	6	
	High rate of relapse	0	2	
	Lack of clinical relevance/standard of care	8	1	
	Data collection complete	1	0	
	Withdrawal of emergency use authorization	0	1	
	Scientific (unknown or unclear)	0	1	
Strategic or business decisions	Corporate Business Decision and Change	19	0	19
Unknown or unclear	Unknown or unclear	7	22	29
Total reasons				197

### Clinical trial transparency, data sharing and disclosure practices

Of the 1,863 studies examined, only 572 (30.70%) posted the results of the clinical trials; 297 publicly provided either study protocol, statistical analysis plan (SAP), or informed consent. 278 studies had study protocols, 270 had statistical analysis plans, and only 64 had consent forms publicly available. 223 trials had study protocol and statistical analysis plan but no consent form while only 45 studies submitted all three. Of the trials that are in Phase 2 or beyond and interventional, 111 trials submitted a study protocol, 107 submitted an SAP, and 28 made an informed consent form publicly available.

## Discussion

This analysis of 1,863 trials with pediatric enrollment, representing10.9% of all mAbs trials registered on ClinicalTrials.gov, highlights important findings related to substantial geographic and income-based concentration, imprecise age inclusion and exclusion, divergent termination patterns by sponsor type, and limited trial transparency.

The geographic distribution of pediatric mAb trials was heavily skewed toward high-income regions. Europe and Central Asia and North America together dominated the trial landscape, while sub-Saharan Africa and South Asia, despite being home to the largest shares of the global pediatric population (22), were the most underrepresented (24). The United States alone contributed five times more trials than the next country, Canada.

The income-based disparity was particularly stark. In terms of location, high-income countries conducted more than seven times the number of trials compared to upper-middle-income countries, and approximately 202 times more trials than low-income countries. Beyond the numerical gap, this geographic concentration raises questions about whose health needs are prioritized in mAb research and development.

Even within the subset of multi-country trials, 66.9% (238/356) were conducted exclusively within countries of the same income tier, indicating that cross-national collaboration in pediatric mAbs research does not meaningfully bridge the gap between high- and low-income settings. Only five trials included no high-income country among their sites; however, the sponsors of these five trials were based in the HICs (Table 1). Notably, fewer than 1% governments (excluding United States) funded pediatric mAbs clinical trials. Of the 17 countries that funded pediatric mAbs trials, only 7 trials were funded by from upper-middle and lower-middle income countries. This disparity is concerning given that LMICs bear a disproportionate share of the global pediatric disease burden. To ensure that the health needs of children from LMICs are addressed would require efforts to diversify funding sources, strengthen international research partnerships, and incentives to ensure that the benefits of mAbs are equitably developed (Table 5) (25). Most of the countries with high trial counts had high representation even in multi-country trials. Notably, China, despite ranking fifth in overall trial count, contributed only 16 multi-country trials, suggesting limited engagement in multi-country trials.

**Table 5:** Opportunities for strengthening the pediatric monoclonal antibody clinical trial landscape: recommendations for stakeholders.

National Library of Medicine (NLM)/National Institutes of Health (NIH)	Policymakers	Funders and sponsors	Civil society	National governments
Transparency & accountability				
Mandate submission of protocol, SAP & consent forms for all pediatric trials. Flag non-compliant records visibly in the database.	Strengthen enforcement of FDAAA 801 & 42 CFR Part 11. Make the non-compliance notices public.	Sponsors should provide greater details regarding the age inclusion/exclusion criteria, rationale for termination of trials, choice of geography, etc. Funders should require protocol & SAP submission as a condition of funding.	Monitor the databases to flag lack of transparency and to hold stakeholders accountable.	Harmonized transparency standards and accountability mechanisms in national regulatory frameworks to ensure greater transparency and accountability.
Geographic & income equity				
	Develop strong networks to incentivize LMIC site inclusion in trials of globally relevant therapies.	Actively design multi-country trials to include substantive LMIC participation. Examine whether research portfolios address infectious disease burden in LMICs. Pursue cross-institutional collaborations to expand geographic reach.	Monitor whether approved mAbs reach LMIC markets and whether evidence is contextually applicable. Advocate for LMIC representation in trial design.	HIC governments: require international site inclusion as a condition of public research funding. Other governments: invest in trial infrastructure. Both: Engage and develop global trial networks.
Pediatric-specific trial design				
Add questions which highlight why specific trials include the pediatric population. Require minimum and maximum age entry at registration or explanation if not added.	Expand the scope of RACE Act to encompass mAbs across all therapeutic areas, not only oncology.	Fund trials with clearly defined pediatric subgroup stratification or pediatric research plans. Require reporting of age-specific outcomes. Avoid enrolling children as marginal subsets of adult trials.	Advocate for pediatric-relevant trial design in regulatory consultations.	Embed pediatric-specific design standards in national clinical trial ethics and regulatory guidelines.
Trial completion & termination				
Mandate structured termination reason reporting using a controlled vocabulary. Track and publish termination patterns annually by sponsor type and disease area.	Introduce regulatory requirements for sponsors to report and justify early termination, particularly for pediatric trials where evidence gaps are critical.	For industry: explore handoff mechanisms after commercially driven termination through technology transfer & knowledge management to CDMOs/new partners Engage patient communities early to mitigate low accrual risk. Work with stakeholders to pursue mixed-funding partnerships combining industry, NIH, national governments, and philanthropic sources to reduce dependence on single sponsor type and shield trials from commercial discontinuation. Develop advanced market commitments and other push incentives to reduce termination of trials on any	Track terminated trials and advocate for handoff mechanisms so halted pediatric trials can be resumed by academic or public partners.	Identify systemic causes limiting pediatric trials.
Sponsor & funder responsibility				
Clarify and publish definitions for all funder type categories, including “Network.” Conduct periodic audits of sponsor/funder classification accuracy.	Design incentive structures for sponsors who conduct LMIC-inclusive pediatric mAbs trials or address under-researched disease areas.	Fund dedicated recruitment & retention infrastructure alongside trial budgets to reduce accrual failures. Avoid pediatric programs de-prioritization driven by market factors.	Highlight cases where commercial discontinuation leads to missing evidence base to advocate for diversified, public-interest driven funding models for pediatric mAbs research.	Increase national public investment in pediatric mAbs research, particularly for disease burdens not commercially attractive to industry.

The majority of pediatric mAbs trials were funded by non-industry, non-U.S. government NIH entities categorized as “Other” (60.1%), which includes universities, academic medical centers, and community organizations. Pediatric mAbs trials funded by industry accounted for 26.36% while those funded by the U.S. government (NIH and other U.S. federal agencies) accounted for 9.45% of all trials. The dominance of academic and institutional sponsors is reflected in the top sponsors list, where cancer centers such as M.D. Anderson (*n* = 109) and Memorial Sloan Kettering (*n* = 51) led alongside NIH institutes. This concentration of sponsorship within oncology-focused institutions aligns with the prominence of cancer-related compounds in the dataset. The most frequently studied compounds included rituximab (*n* = 284), bevacizumab (*n* = 155), alemtuzumab (*n* = 130), nivolumab (*n* = 102), ipilimumab (*n* = 49), brentuximab vedotin (*n* = 49), and cetuximab (*n* = 36).

When looking at trials focusing on infectious diseases, our study identified 168 pediatric mAbs clinical trials for infectious diseases spanning five pathogen categories. There is a growing pipeline of novel agents across viral, bacterial, parasitic, and fungal conditions. However, the distribution of trials is markedly uneven. RSV (*n* = 45) and COVID-19 (*n* = 34) account for the majority of clinical trials. The funding landscape across categories is also inconsistent. Industry dominated viral trials overall (*n* = 46, 50%), particularly RSV (62%) while COVID-19 funding was more mixed (industry 41%, other 50%). In contrast, chronic viral trials were predominantly other-funded (58.7%) or U.S. government NIH-funded (26.1%), with minimal industry involvement (6.5%). Parasitic disease trials were entirely non-industry funded (U.S. government NIH 36%, other 64%), suggesting limited industry interest in neglected tropical disease drug development. Parasitic disease trials stand out as a key category where all 11 trials were conducted exclusively in LMIC settings. All 9 malaria trials were conducted in Mali, Kenya, Burkina Faso, Uganda, and Tanzania—all of which are classified among the WHO high-burden countries for malaria (26).

Fungal disease trials were the most sparsely represented disease category, with only three trials classified under fungal conditions—and notably, none involved a mAb targeting an invasive fungal infection. Of the three, one trial (NCT00787917) evaluated omalizumab, a mAb targeting IgE, for allergic bronchopulmonary aspergillosis (ABPA) in the context of cystic fibrosis—an inflammatory complication of fungal colonization rather than an invasive fungal infection. The remaining two trials evaluated conventional antifungal agents in children with granulocytopenia and fever in children with cancer and were captured in this dataset because of monoclonal gammopathy of undetermined significance. These findings indicate that pediatric mAb development for invasive fungal infections is, to our knowledge, essentially nonexistent. This gap is particularly concerning given the clinical burden of pediatric invasive fungal infections e.g., *Candida* and *Aspergillus* (27).

Nearly one in ten pediatric mAb trials (199/1,863, 10.7%) were terminated before completion, and an overwhelming majority of terminated trials were interventional trials (191/199, 96.0%). Thus, the bulk of this loss falls precisely in the category of trials designed to generate the highest quality efficacy and safety data for children. Across all sponsor types, logistic, operational, and administrative reasons constituted the single largest domain of termination (*n* = 103, 52.28%), with low patient accrual, enrollment difficulties, and participant attrition accounting for the majority of cases within this domain (*n* = 75, 38.07%). This pattern reflects a structural challenge intrinsic to pediatric research: eligible populations are smaller, consent processes are more complex, and accrual failure is rarely attributable to a single cause. Studies suggest that effective recruitment and retention of pediatric participants require a multifaceted approach right from study design and community outreach through to consent, incentives, and follow-up (28). Addressing these challenges requires rigorous pre-trial feasibility assessments, innovative trial designs and adaptive statistical methods, and earlier engagement with patient communities to improve recruitment sustainability (28–30). The proportion of terminations attributed to unknown or unclear reasons (*n* = 29, 14.4%) limits the ability of the scientific community to understand why trials were halted and identify systemic patterns leading to terminations. This is particularly critical in the context of pediatric mAbs research, where designing and conducting trials for this key population is challenging and resource-intensive, and where the broader evidence base remains sparse (31).

The divergence in termination patterns by funder type is stark. NIH-sponsored trials were predominantly terminated for logistic and operational reasons (*n* = 39), with low accrual and attrition alone accounting for 27 of these cases. Financial and resource constraints, though less frequent (*n* = 4), were exclusively observed in U.S. government NIH and non-industry trials, with no industry-funded trial reporting funding withdrawal as a reason for termination. This asymmetry likely reflects the different funding structures at play: publicly funded trials are subject to higher terminations due to several challenges including funding (32). Industry-funded terminations, by contrast, were more evenly distributed across scientific (*n* = 20), strategic or business (*n* = 19), and logistic domains (*n* = 18). Notably, corporate business decisions and changes in business priorities represented the most frequent single termination reason among industry trials (*n* = 19), which was absent among U.S. government NIH and other sponsors. This pattern suggests a fundamental vulnerability of commercially driven pediatric research: trials may be discontinued not because of scientific failure or operational difficulty, but because of shifts in corporate strategy, pipeline reprioritization, or changes in the anticipated commercial value of a product.

Scientific reasons contributed meaningfully across both funder types (industry *n* = 20, NIH and others *n* = 19), with insufficient efficacy or unsatisfactory risk-benefit ratios, failure of interim analyses, and safety or toxicity concerns each contributing to terminations. The presence of safety-related terminations (*n* = 9 overall) reflect that some trial terminations represent unavoidable loss.

In terms of age, enrollment of pediatric populations across these trials was imprecise. First, the minimum enrollment age was unspecified in 405 trials. Second, the mean age gap between minimum and maximum enrollment ages was 35.3 years, reflecting considerable heterogeneity in the populations tagged under the “pediatric” label. Further, only 239 of 1,863 trials (12.8%) restricted enrollment exclusively to children (birth-17 years). The majority of clinical trials enrolled children alongside adults (18–64 years) and older adults (older than 65 years)– reflecting trials that permitted pediatric enrollment rather than trials designed primarily for pediatric populations. Therefore, the dataset, with 1,863 studies, reflects the upper bound of pediatric mAb research and findings should be interpreted as trials permitting pediatric enrollment rather than trials exclusively focused on children as the primary study population.

Critically, the pediatric population is not a homogeneous group. FDA recognizes five distinct pediatric age categories for clinical studies: neonates (newborns up to 1 month), infants (1 month–2 years), children (2–12 years), adolescents (12–16 years), and other (33). However, ClinicalTrials.gov does not incorporate these categories into its registry infrastructure, instead collapsing all pediatric participants into a single broad designation of “Child” i.e., birth to 17 years. This disconnect between regulatory classification, and registry design makes it challenging to determine from ClinicalTrials.gov records alone whether a trial enrolled neonates, infants, children, adolescents, and whether the evidence generated is applicable to a specific subgroup or all of them.

Furthermore, a subset of trials enrolling children investigated conditions with no meaningful pediatric relevance. For instance, NCT06058234, NCT00606476, and NCT06530732 focused on Alzheimer's disease, while NCT05884372, NCT01652690, NCT00091793, and NCT00890981 focused on postmenopausal osteoporosis. While these likely reflect errors in age eligibility classification rather than actual enrollment of children in clinically irrelevant trials, they point to important inconsistencies in how pediatric eligibility is recorded in ClinicalTrials.gov. A comprehensive audit of all 1,863 trials for condition-age analysis was beyond the scope of this study. However, these observations suggest that addition of “child” as an age classification may overestimate the extent to which children are represented as the focus population likely falls below the estimated 10.9%.

There continues to be limited research on pediatric mAbs despite a progressive regulatory landscape that requires pediatric-specific clinical evidence. The Pediatric Research Equity Act (PREA, 2003) granted the FDA authority to require pediatric studies for drugs and biologics and, under Section 505B(d)(1) of the Federal Food, Drug, and Cosmetic Act, empowered the agency to require sponsors to submit a pediatric assessment for molecularly targeted cancer investigations, with non-compliance notices available as an enforcement mechanism (34). Building on this, the Research to Accelerate Cures and Equity (RACE) for Children Act, enacted as Title V, Section 504 of the FDA Reauthorization Act in August 2017, further expanded mandatory pediatric evaluation of new molecularly targeted drugs and biologics intended for adult cancers where the molecular target is substantially relevant to the growth or progression of a pediatric cancer; thereby shifting the criteria for pediatric assessment from clinical indication to mechanism of action (35). However, these regulatory advances remain limited in their scope. Both PREA and RACE's mandate is limited to pediatric cancers, leaving the rapidly expanding non-oncology applications of mAbs, particularly autoimmune diseases, infectious diseases, and rare conditions, outside its reach. This is a critical gap.

Although ClinicalTrials.gov was launched in 2000, following the passage of the Food and Drug Administration Modernization Act of 1997 under Section 113 which directed the creation of a public registry of clinical trials for “serious or life-threatening diseases and conditions”, increased calls for clinical trial transparency continued in the early 2000s due to significant concerns over lack of full disclosure of results including safety data and negative trial results and to increase public trust (36, 37). Subsequently, Section 801 of the Food and Drug Administration Amendments Act of 2007 (FDAAA 801) enforced ethical accountability in clinical research by requiring the registration of clinical trials on ClinicalTrials.gov (38). The implications of lack of clinical trial transparency are particularly acute in the context of pediatric mAbs research where the evidence is sparse, the population vulnerable, trials precarious, and the consequences of error higher. Low transparency impedes the ability of the scientific community to identify methodological weaknesses, assess whether trial designs are appropriate for pediatric subpopulations, and build on prior evidence. It also limits accountability: without public access to protocols and statistical analysis plans (SAPs), it is difficult to assess whether trials were conducted as planned, whether outcomes were selectively reported, or whether age eligibility criteria were applied consistently.

The findings from our analysis reveal a substantial transparency gap in the pediatric mAbs trial landscape. Of the 1,863 trials, only 572 posted the results and 297 publicly shared either study protocol, SAP, or informed consent form and only 45 submitted all three. When the analysis is restricted to trials subject to mandatory disclosure requirements under the Final Rule for Clinical Trials Registration and Results Information Submission (42 CFR Part 11)—interventional trials of Phase 2 and above registered after January 2017, compliance still remains strikingly low: only 111 trials submitted a study protocol, 107 submitted an SAP, and just 28 made an informed consent form publicly available (39). These findings are consistent with, and reinforce, broader evidence of systemic non-compliance with FDAAA 801 reporting requirements and 42 CFR Part 11 wherein approximately 60% of applicable trials fail to report results on time, and that among industry-sponsored trials, fewer than half fully comply with FDAAA results reporting mandates (40). Additional studies have also reported significant delays in reporting results of pediatric clinical trials, reiterating the need for enhanced compliance in result reporting (41). The informed consent gap is especially troubling; of the filtered subset of trials with mandatory disclosure obligations, only 28 made consent forms publicly available. Informed consent documents are a critical window into how trial risks and benefits were communicated to families and caregivers, and their absence from the public record undermines accountability for the ethical conduct of research involving children. This is particularly relevant given the longstanding ethical complexities of pediatric trial enrollment, where consent is provided by parents or guardians on behalf of participants who cannot themselves authorize participation.

At the registry level, NIH should strengthen enforcement mechanisms, embedding transparency requirements directly into the ClinicalTrials.gov interface by visibly flagging records where mandatory documents are missing or overdue and should use their power FDAAA 801 to identify non-compliant trials rather than waiting for FDA to act first (38). The FDA, building on its precedent of issuing Notices of Noncompliance under FDAAA 801, should ensure this enforcement is consistently and proactively applied (40). Funders, both public and private, should condition trial approvals and grant awards on timely submission of protocols, SAPs, and consent forms. Without such systematic enforcement, the transparency infrastructure surrounding pediatric mAbs trials will continue to fall short of ensuring that research conducted is scientifically sound, embedded with principles of transparency and accountability.

### Limitations

First, as a landscape analysis, the study is intentionally descriptive. To our knowledge, it is the first such analysis of the global pediatric mAb clinical trial landscape and establishes a baseline for accountability and future analytical and policy-oriented research.

Second, this analysis is restricted to ClinicalTrials.gov and does not capture trials registered exclusively in country-specific registries or the WHO International Clinical Trials Registry Platform (ICTRP). While ClinicalTrials.gov is the largest clinical trials registry globally, this scope decision was made deliberately to avoid the methodological challenges of merging heterogeneous data sources and the risk of duplicate trial records across registries. As a result, the geographic distribution of trials reported here may underestimate research activity in regions where national registries are the predominant registration platform.

## Conclusion

Our cross-sectional analysis of 1,863 mAbs clinical trials registered on ClinicalTrials.gov that permitted pediatric enrollment reveals a research landscape that is geographically concentrated: trials in high-income countries were conducted more than 200 times than in lower-income countries, and even multi-country collaborations rarely bring together high- and low-income settings.

The funding architecture of pediatric mAbs research reflects structural imbalances. Academic and institutional funders, classified under “Other,” drive the majority of trials, concentrated heavily in oncology. This pattern mirrors broader trends in pediatric drug development: Bourgeois et al. found that governmental and non-profit organizations funded 58.6% of pediatric trials compared with 35.0% of adult trials, underscoring the longstanding dependence of pediatric research on public and philanthropic rather than commercial funding (10).

Trial attrition represents a further systemic concern, with low patient accrual as the leading cause across all sponsor types, and commercially driven discontinuation a distinctive pattern among industry-funded trials. Compounding these challenges is the transparency deficit. Fewer than a third of trials posted results, and only a small fraction made study protocols, statistical analysis plans, and informed consent forms publicly available despite the mandatory disclosure requirements. This gap undermines scientific accountability in pediatric research.

Taken together, these findings call for coordinated action across regulatory agencies, funders, sponsors, national governments, and civil society (Table 5). Strengthening enforcement of transparency requirements, expanding the geographic and income diversity of trial sites, broadening the regulatory mandate for pediatric evaluation beyond oncology, and building more resilient, mixed-funding models for pediatric research are all essential steps.

## Data Availability

The original contributions presented in the study are included in the article/Supplementary Material, further inquiries can be directed to the corresponding author.
