# Supplementary material for: The global landscape of pediatric monoclonal antibody (mAb) clinical trials: an analysis of ClinicalTrials.gov
Source: Front Pediatr. 2026 Sep 1;14:1846142. doi: 10.3389/fped.2026.1846142 (PMC13574800; doi:10.3389/fped.2026.1846142)
Supplement: Supplementary file 1 [file Table3.docx]

library(readxl)

library(tidyr)

library(stringr)

library(ggplot2)

library(lubridate)

library(sf)

library(rnaturalearth)

library(rnaturalearthdata)

library(openxlsx)

library(dplyr)

library(knitr)

library(kableExtra)

library(ggthemes)

library(stringi)

library(tibble)

setwd("/Users/agratasharma/Desktop")

trials_file <- "mabs_peds.xlsx"

wb_file	<- "world_bank_classification.xlsx"

bar_with_labels <- function(data, x_var, y_var) {

ggplot(data, aes(x = {{ x_var }}, y = {{ y_var }})) +

geom_col(fill = "steelblue") +

geom_text(aes(label = {{ y_var }}), vjust = -0.3, size = 4) +

theme_classic(base_size = 13)

}

norm_key <- function(x) {

x %>%

as.character() %>%

str_replace_all("[’`´]", "'") %>%

stringi::stri_trans_general("Latin-ASCII") %>%

str_squish()

}

data_1 <- read_excel(trials_file)

data_1 <- data_1 %>%

mutate(

Trial_ID = as.character(`NCT Number`),

Locations = as.character(Locations)

) %>%

filter(!is.na(Trial_ID), str_trim(Trial_ID) != "") %>%

mutate(

Locations = str_replace_all(Locations, '[\r\n"]', ""),

Locations = str_replace_all(Locations, "\\.$", ""),

Locations = str_replace_all(Locations, "Korea, Republic of", "South Korea"),

Locations = str_replace_all(Locations, "Iran, Islamic Republic of", "Iran"),

Locations = str_replace_all(Locations, "Moldova, Republic of", "Moldova"),

Locations = str_replace_all(Locations, "Congo, The Democratic Republic of the", "Democratic Republic of Congo")

)

trial_countries <- data_1 %>%

filter(!is.na(Locations), str_trim(Locations) != "") %>%

separate_rows(Locations, sep = "\\|") %>%

mutate(

has_comma = str_detect(Locations, ","),

country_field = if_else(

has_comma,

str_trim(str_extract(Locations, "[^,]+$")),

str_trim(Locations)

)

) %>%

separate_rows(country_field, sep = ",") %>%

mutate(country = str_squish(str_trim(country_field))) %>%

filter(!is.na(country), country != "") %>%

distinct(Trial_ID, country)

trial_country_summary <- trial_countries %>%

group_by(Trial_ID) %>%

summarise(

n_countries	= n_distinct(country),

country	= paste(sort(unique(country)), collapse = ", "),

multi_country = if_else(n_countries > 1, "Multi-Country", "Single-Country"),

.groups = "drop"

)

data_1 <- data_1 %>%

select(-any_of(c("n_countries", "country", "multi_country",

"multi_country.x", "multi_country.y"))) %>%

left_join(trial_country_summary, by = "Trial_ID")

trial_country_summary %>% count(multi_country)

\# ----------------------------------------------------------

map_name_fix <- c(

"United States"	= "United States of America",

"Russian Federation" = "Russia"

)

wb_map <- c(

"United States of America" = "United States",

"Russia"	= "Russian Federation",

"South Korea"	= "Korea, Rep.",

"North Korea"	= "Korea, Dem. People's Rep.",

"Iran"	= "Iran, Islamic Rep.",

"Egypt"	= "Egypt, Arab Rep.",

"Turkey (Türkiye)"	= "Turkiye",

"Turkey"	= "Turkiye",

"Slovakia"	= "Slovak Republic",

"Taiwan"	= "Taiwan, China",

"Hong Kong"	= "Hong Kong SAR, China",

"Venezuela"	= "Venezuela, RB",

"Vietnam"	= "Viet Nam",

"Côte d’Ivoire"	= "Cote d'Ivoire",

"Côte d'Ivoire"	= "Cote d'Ivoire",

"Ivory Coast"	= "Cote d'Ivoire",

"Democratic Republic of Congo"	= "Congo, Dem. Rep.",

"Democratic Republic of the Congo" = "Congo, Dem. Rep.",

"Republic of the Congo"	= "Congo, Rep."

)

trials_per_country <- trial_countries %>%

distinct(Trial_ID, country) %>%

count(country, name = "Num_Trials") %>%

mutate(country_for_map = recode(country, !!!map_name_fix, .default = country))

world <- ne_countries(scale = "medium", returnclass = "sf")

merged_map <- world %>%

left_join(trials_per_country, by = c("name" = "country_for_map"))

ggplot(merged_map) +

geom_sf(aes(fill = Num_Trials), color = "black", size = 0.1) +

scale_fill_gradient(

low = "#deebf7",

high = "#08519c",

trans = "log10",

breaks = c(1, 10, 100, 1000),

labels = c("1", "10", "100", "1000"),

na.value = "lightgray",

name = "Trials"

) +

labs(title = "Number of clinical trials by country") +

theme_map()

\# ----------------------------------------------------------

\# PART 2 — World Bank join + region/income charts

\# ----------------------------------------------------------

wb_data <- read_excel(wb_file) %>%

mutate(Economy_key = norm_key(Economy))

\# Add Venezuela if missing

if (!any(wb_data$Economy_key == norm_key("Venezuela, RB"))) {

wb_data <- bind_rows(

wb_data,

tibble(

Economy = "Venezuela, RB",

Region = "Latin America & Caribbean",

`Income group` = "Unclassified"

)

) %>%

mutate(Economy_key = norm_key(Economy))

}

merged_wb <- trials_per_country %>%

transmute(Country = country_for_map, Num_Trials) %>%

mutate(

Country_WB = recode(Country, !!!wb_map, .default = Country),

Country_WB_key = norm_key(Country_WB)

) %>%

left_join(wb_data, by = c("Country_WB_key" = "Economy_key"))

write.xlsx(merged_wb, "merged_country_worldbank.xlsx")

region_trials <- merged_wb %>%

filter(!is.na(Region)) %>%

group_by(Region) %>%

summarise(Total_Trials = sum(Num_Trials, na.rm = TRUE), .groups = "drop")

bar_with_labels(region_trials,

reorder(Region, -Total_Trials),

Total_Trials) +

labs(

title = "Number of trials by region (WB matched)",

x = "Region",

y = "Number of trials"

) +

theme(axis.text.x = element_text(angle = 45, hjust = 1))

income_trials <- merged_wb %>%

filter(!is.na(`Income group`)) %>%

group_by(`Income group`) %>%

summarise(Total_Trials = sum(Num_Trials, na.rm = TRUE), .groups = "drop")

bar_with_labels(income_trials,

reorder(`Income group`, -Total_Trials),

Total_Trials) +

labs(

title = "Number of trials by income group (WB matched)",

x = "Income group",

y = "Number of trials"

) +

theme(axis.text.x = element_text(angle = 45, hjust = 1))

\# ----------------------------------------------------------

data_1 <- data_1 %>%

mutate(

StartDate_raw = str_trim(as.character(`Start Date`)),

StartDate_parsed = suppressWarnings(parse_date_time(

StartDate_raw,

orders = c("Ymd", "Y-m-d", "Y-m", "Y", "m/d/Y", "d/m/Y", "mdY", "dmY", "b Y", "B Y")

)),

Year = year(StartDate_parsed)

)

multi_country_long <- data_1 %>%

distinct(Trial_ID, Year, multi_country) %>%

filter(multi_country == "Multi-Country") %>%

left_join(trial_countries %>% distinct(Trial_ID, country), by = "Trial_ID") %>%

mutate(

country_for_map = recode(country, !!!map_name_fix, .default = country),

country_wb = recode(country_for_map, !!!wb_map, .default = country_for_map),

country_wb_key = norm_key(country_wb)

) %>%

left_join(wb_data, by = c("country_wb_key" = "Economy_key"))

multi_economic_trials <- multi_country_long %>%

group_by(Trial_ID) %>%

summarise(

Income_nonNA = sum(!is.na(`Income group`)),

Economic_Groups = n_distinct(`Income group`, na.rm = TRUE),

Mixed_Economic = case_when(

Economic_Groups > 1 ~ "Yes",

TRUE	~ "No"

),

.groups = "drop"

)

mixed_economic_summary <- multi_economic_trials %>%

count(Mixed_Economic, name = "Count")

ggplot(mixed_economic_summary, aes(x = "", y = Count, fill = Mixed_Economic)) +

geom_col(width = 1) +

coord_polar("y") +

labs(

title = "Proportion of multi-country trials with mixed economic groups",

fill = "Mixed economic"

) +

theme_void()

mixed_economic_summary <- multi_economic_trials %>%

filter(Mixed_Economic != "Unknown") %>%

count(Mixed_Economic, name = "Count") %>%

mutate(

Percent = round(Count / sum(Count) * 100, 1),

Label = paste0(Count, " (", Percent, "%)")

)

ggplot(mixed_economic_summary, aes(x = "", y = Count, fill = Mixed_Economic)) +

geom_col(width = 1) +

coord_polar("y") +

geom_text(

aes(label = Label),

position = position_stack(vjust = 0.5),

size = 4

) +

labs(

title = "Multi-country trials by income composition",

fill = "Income composition"

) +

theme_void()

ggplot(mixed_economic_summary,

aes(x = Mixed_Economic, y = Count)) +

geom_col(fill = "steelblue") +

geom_text(aes(label = paste0(Percent, "%")), vjust = -0.3) +

labs(

title = "Multi-country trials with mixed income groups",

x = "",

y = "Number of trials"

) +

theme_classic()

yearly_summary <- data_1 %>%

filter(multi_country == "Multi-Country", !is.na(Year)) %>%

distinct(Trial_ID, Year) %>%

left_join(multi_economic_trials %>% select(Trial_ID, Mixed_Economic), by = "Trial_ID") %>%

count(Year, Mixed_Economic, name = "Count")

ggplot(yearly_summary,

aes(x = Year, y = Count, color = Mixed_Economic, group = Mixed_Economic)) +

geom_line(linewidth = 1) +

geom_point(size = 2) +

labs(

title = "Multi-country trials over time (mixed economic groups)",

x = "Year",

y = "Number of trials",

color = "Mixed economic"

) +

theme_minimal()

\# ----------------------------------------------------------

trials_hic_status <- multi_country_long %>%

group_by(Trial_ID) %>%

summarise(

Income_nonNA = sum(!is.na(`Income group`)),

HIC_Present = any(`Income group` == "High income", na.rm = TRUE),

Without_HIC = case_when(

Income_nonNA == 0 ~ "Unknown",

HIC_Present	~ "No",

TRUE	~ "Yes"

),

.groups = "drop"

)

hic_status_summary <- trials_hic_status %>%

count(Without_HIC, name = "Count")

bar_with_labels(hic_status_summary, Without_HIC, Count) +

labs(

title = "Multi-country trials without HIC involvement",

x = "Without HIC",

y = "Number of trials"

)

trials_without_hic_yearly <- data_1 %>%

filter(multi_country == "Multi-Country", !is.na(Year)) %>%

distinct(Trial_ID, Year) %>%

left_join(trials_hic_status %>% select(Trial_ID, Without_HIC), by = "Trial_ID") %>%

filter(Without_HIC == "Yes") %>%

count(Year, name = "Num_Trials")

\# ----------------------------------------------------------

\# PART 7 — Map of countries in multi-country trials

\# ----------------------------------------------------------

multicountry_map_counts <- trial_countries %>%

left_join(trial_country_summary %>% select(Trial_ID, multi_country), by = "Trial_ID") %>%

filter(multi_country == "Multi-Country") %>%

distinct(Trial_ID, country) %>%

count(country, name = "Num_MultiCountry_Trials") %>%

mutate(country_for_map = recode(country, !!!map_name_fix, .default = country))

merged_multi_map <- world %>%

left_join(multicountry_map_counts, by = c("name" = "country_for_map"))

ggplot(merged_multi_map) +

geom_sf(aes(fill = Num_MultiCountry_Trials), color = "black", size = 0.1) +

scale_fill_gradientn(

colors = c("lightblue", "blue", "darkblue"),

na.value = "lightgray",

guide = "colorbar"

) +

labs(

title = "Countries participating in multi-country trials",

fill = "Multi-country\ntrials"

) +

theme_map()

\# ----------------------------------------------------------

unmatched_wb <- merged_wb %>%

filter(is.na(Region) | is.na(`Income group`)) %>%

arrange(desc(Num_Trials)) %>%

select(Country, Country_WB, Num_Trials)

if (nrow(unmatched_wb) > 0) {

message("WARNING: Unmatched WB join (top 30):")

print(head(unmatched_wb, 30))

}

multi_country_long %>%

group_by(Trial_ID) %>%

summarise(all_income_missing = all(is.na(`Income group`)), .groups = "drop") %>%

count(all_income_missing)

multi_country_long %>%

summarise(

total_rows = n(),

missing_income_rows = sum(is.na(`Income group`))

)

multi_country_long %>%

group_by(Trial_ID) %>%

summarise(

n_countries = n(),

n_income_missing = sum(is.na(`Income group`)),

.groups = "drop"

) %>%

filter(n_income_missing > 0) %>%

head(10)

multi_no_hic_table <- multi_country_long %>%

left_join(trials_hic_status %>% select(Trial_ID, Without_HIC), by = "Trial_ID") %>%

filter(Without_HIC == "Yes") %>%

group_by(Trial_ID, Year) %>%

summarise(

Countries = paste(sort(unique(country)), collapse = ", "),

Income_Groups = paste(sort(unique(`Income group`[!is.na(`Income group`)])), collapse = ", "),

.groups = "drop"

) %>%

arrange(desc(Year))

View(multi_no_hic_table)

multi_no_hic_table <- multi_country_long %>%

left_join(trials_hic_status %>% select(Trial_ID, Without_HIC), by = "Trial_ID") %>%

filter(Without_HIC == "Yes") %>%

group_by(Trial_ID, Year) %>%

summarise(

n_countries = n_distinct(country), # count of countries

Countries = paste(sort(unique(country)), collapse = ", "),

Income_Groups = paste(sort(unique(`Income group`[!is.na(`Income group`)])), collapse = ", "),

.groups = "drop"

) %>%

arrange(desc(Year), desc(n_countries))

trials_per_country_table <- trial_countries %>%

distinct(Trial_ID, country) %>%	# ensures no duplicates

count(country, name = "Num_Trials") %>%

arrange(desc(Num_Trials))

View(trials_per_country_table)

install.packages("stargazer")

library(stargazer)

View(trials_per_country_table)

stargazer(trials_per_country_table, type = "text")

stargazer(

trials_per_country_table,

type = "text",

title = "Number of Trials per Country",

summary = FALSE,

rownames = FALSE

)

stargazer(

trials_per_country_table %>% slice(1:20),

type = "text",

title = "Top 20 Countries by Number of Trials",

summary = FALSE,

rownames = FALSE

)

summary_counts <- trial_country_summary %>%

count(multi_country) %>%

mutate(

Percent = round(n / sum(n) * 100, 1)

)

summary_counts

mixed_summary <- multi_economic_trials %>%

count(Mixed_Economic, name = "Count") %>%

mutate(

Percent = round(Count / sum(Count) * 100, 1)

)

mixed_summary

conditions_2020_2025 <- data_1 %>%

filter(

multi_country == "Multi-Country",

Year >= 2020,

Year <= 2025

) %>%

select(Trial_ID, Year, Conditions) %>%

distinct()

conditions_2020_2025_long <- conditions_2020_2025 %>%

separate_rows(Conditions, sep = ",") %>%

mutate(Condition = stringr::str_squish(Conditions)) %>%

filter(!is.na(Condition), Condition != "")

condition_trends <- conditions_2020_2025_long %>%

count(Year, Condition, name = "Num_Trials") %>%

arrange(desc(Num_Trials))

top_conditions <- conditions_2020_2025_long %>%

count(Condition, name = "Total_Trials") %>%

arrange(desc(Total_Trials)) %>%

slice(1:10)

income_country_trend <- country_year_income %>%

filter(Year <= 2025) %>%

count(Year, `Income group`, name = "Num_Countries")

country_year_income <- trial_countries %>%

left_join(data_1 %>% select(Trial_ID, Year), by = "Trial_ID") %>%

mutate(

country_for_map = recode(country, !!!map_name_fix, .default = country),

country_wb = recode(country_for_map, !!!wb_map, .default = country_for_map),

country_wb_key = norm_key(country_wb)

) %>%

left_join(wb_data, by = c("country_wb_key" = "Economy_key")) %>%

filter(!is.na(Year), !is.na(`Income group`))

income_country_trend <- country_year_income %>%

count(Year, `Income group`, name = "Num_Countries")

income_country_trend <- income_country_trend %>%

mutate(

Income_group_short = case_when(

`Income group` == "High income" ~ "HIC",

`Income group` == "Upper middle income" ~ "UMIC",

`Income group` == "Lower middle income" ~ "LMIC",

`Income group` == "Low income" ~ "LIC",

TRUE ~ "Other"

)

)

ggplot(income_country_trend,

aes(x = Year, y = Num_Countries, color = Income_group_short)) +

geom_line(linewidth = 1) +

geom_point(size = 2) +

labs(

title = "Country participation in trials by income group over time",

x = "Year",

y = "Number of country participations",

color = "Income group"

) +

theme_minimal()

income_country_pct <- country_year_income %>%

filter(Year <= 2025) %>%	# ✅ removes 2026

count(Year, `Income group`) %>%

group_by(Year) %>%

mutate(

Percent = n / sum(n) * 100,

Income_group_short = case_when(

`Income group` == "High income" ~ "HIC",

`Income group` == "Upper middle income" ~ "UMIC",

`Income group` == "Lower middle income" ~ "LMIC",

`Income group` == "Low income" ~ "LIC",

TRUE ~ "Other"

)

)

income_country_pct <- country_year_income %>%

filter(Year <= 2025) %>%

count(Year, `Income group`) %>%

group_by(Year) %>%

mutate(

Percent = n / sum(n) * 100,

Income_group_short = case_when(

`Income group` == "High income" ~ "HIC",

`Income group` == "Upper middle income" ~ "UMIC",

`Income group` == "Lower middle income" ~ "LMIC",

`Income group` == "Low income" ~ "LIC",

TRUE ~ "Other"

)

)

income_country_pct <- country_year_income %>%

count(Year, `Income group`) %>%

group_by(Year) %>%

mutate(

Percent = n / sum(n) * 100,

Income_group_short = case_when(

`Income group` == "High income" ~ "HIC",

`Income group` == "Upper middle income" ~ "UMIC",

`Income group` == "Lower middle income" ~ "LMIC",

`Income group` == "Low income" ~ "LIC",

TRUE ~ "Other"

)

)

ggplot(income_country_pct,

aes(x = Year, y = Percent, fill = Income_group_short)) +

geom_area() +

labs(

title = "Share of country participation in trials by income group",

x = "Year",

y = "Percent"

) +

theme_minimal()

country_year_income <- trial_countries %>%

left_join(data_1 %>% select(Trial_ID, Year), by = "Trial_ID") %>%

mutate(

country_for_map = recode(country, !!!map_name_fix, .default = country),

country_wb = recode(country_for_map, !!!wb_map, .default = country_for_map),

country_wb_key = norm_key(country_wb)

) %>%

left_join(wb_data, by = c("country_wb_key" = "Economy_key")) %>%

filter(!is.na(Year), !is.na(`Income group`))

country_year_income %>%

filter(Year <= 2025)

phase_income_table <- phase_income_combo %>%

distinct(Trial_ID, Phase_Combo, Income_Short) %>%

count(Phase_Combo, Income_Short, name = "Num_Trials") %>%

pivot_wider(

names_from = Income_Short,

values_from = Num_Trials,

values_fill = list(Num_Trials = 0)

) %>%

mutate(

HIC = coalesce(HIC, 0),

UMIC = coalesce(UMIC, 0),

LMIC = coalesce(LMIC, 0),

LIC = coalesce(LIC, 0)

) %>%

select(Phase_Combo, HIC, UMIC, LMIC, LIC) %>%

arrange(desc(HIC + UMIC + LMIC + LIC))
